# The Number and Size of Invasion Areas in Mixed-Type Carcinoma in Female Dogs Are Associated with Regional Metastases

**DOI:** 10.3390/vetsci12040318

**Published:** 2025-03-31

**Authors:** Fernanda Freitas Miranda, Ana Paula Vargas Garcia, Marina Possa dos Reys, Erica Almeida Viscone, Mayra Cunha Flecher, Michele Angela Rodrigues, Geovanni Dantas Cassali

**Affiliations:** 1 Department of General Pathology, Institute of Biological Sciences, Federal University of Minas Gerais, Belo Horizonte 31270-901, MG, Brazil; fernandafreitas@vetufmg.edu.br (F.F.M.); apvg@ufmg.br (A.P.V.G.); marinareys@ufmg.br (M.P.d.R.); michele.rodrigues@yale.edu (M.A.R.); 2 Veterinary Diagnostic Center, Celulavet, Belo Horizonte 31365-000, MG, Brazil; ericaviscone@gmail.com; 3 Department of Veterinary Medicine, University of Vila Velha, Vila Velha 45570-000, ES, Brazil; mayra.flecher@uvv.br

**Keywords:** mammary gland, tumor invasion, lymph node, metastasis, tumoral stroma

## Abstract

Mixed-type carcinoma (MTC) accounts for 44% of mammary neoplasms in bitches and poses challenges in histological grading due to the heterogeneity of invasive areas. The present study aimed to measure and characterize invasive areas in the stroma of MTC and investigate the association of those parameters with lymph node status and survival. Forty cases were analyzed and divided into two groups: with metastasis and without metastasis. The size, perimeter, length, and number of invasive carcinomatous areas were measured. Cases with metastasis showed a higher number of foci (*p* = 0.003) and larger invasive areas (*p* = 0.006) compared to cases without metastasis. Bitches with invasive areas larger than 2 mm^2^ had shorter survival times. No association was found between the perimeter or length of invasive areas, the expression of Ki67, ER, PR, COX-2, HER-2, and the immunophenotype. Thus, the number and size of invasive areas are associated with regional lymph node status, suggesting histomorphometry may serve as a valuable tool for evaluating the biological behavior of mixed-type carcinoma.

## Simple Summary

Mammary tumors in female dogs can be heterogeneous and difficult to classify. This study looked at specific features of cancer spread in these tumors to see if they could predict how aggressive the cancer would be. By measuring the size and number of cancer invasion areas, researchers found that tumors with more and larger invasion areas were more likely to spread to lymph nodes and were linked to shorter survival times in dogs. These findings suggest that measuring these features could help veterinarians better understand and predict the behavior of these heterogeneous mammary tumors.

## 1. Introduction

Mixed tumors are among the most frequently diagnosed neoplasms in the mammary gland of bitches, with a predominance of mixed-type carcinoma (MTC) [1], also referred to as carcinomas in mixed tumors [2]. A comprehensive epidemiological study involving 1539 cases of canine mammary tumors revealed that 85% of the lesions were malignant, while only 13% were benign. Among the histological subtypes, MTC was the most prevalent, accounting for 44% of the analyzed neoplasms [3].

MTCs differ from benign mixed tumors by the presence of malignant epithelial cells. This type of carcinoma is characterized by the coexistence of benign mesenchymal components, including cartilage, bone, and adipose tissue [1,2,4,5]. The development of MTC arises from the malignant transformation of epithelial cells present in benign mixed tumors. During this progression, areas of carcinoma in situ can be observed, which eventually evolve into invasive forms [2,6]. The in situ phase is characterized by malignant epithelial proliferation occupying the ductal lumen, while the myoepithelial cell layer and the integrity of the basement membrane are preserved [7,8,9,10].

Microinvasion is defined as the presence of carcinoma foci in situ with microscopic peripheral areas of neoplastic cells, measuring 1 mm in diameter or less, that extend beyond the basement membrane [11,12,13]. In invasive areas, the continuity of the myoepithelial layer and basement membrane is lost, allowing malignant epithelial cells to infiltrate the stroma, with eventual replacement of the pre-existing benign lesion [13,14,15]. Such invasive areas in mammary carcinomas are classified according to the Nottingham system, as modified by Elston and Ellis (1991) [16]. However, in MTC, this classification is not always feasible due to the absence of 10 distinct invasive areas in high-power fields (HPFs) [17,18]. According to Nunes (2019) [5], only 12% of the 682 MTC cases analyzed presented sufficient invasive areas for histological classification, highlighting a common diagnostic limitation in this type of neoplasm.

MTCs are the most frequently diagnosed neoplasms in the canine mammary gland and generally present with a favorable prognosis. However, specific cases may progress to regional or distant metastases, leading to a reduced survival time and therefore requiring appropriate therapeutic interventions [2,3,5,6,7,8,11,12]. In this context, the present study aims to evaluate epithelial areas in the stroma and investigate their association with the occurrence of regional metastases, as well as with patient survival.

## 2. Materials and Methods

### 2.1. Ethical Aspects

The study was carried out according to ethical principles for the use of animals in experimentation and was approved by the Ethics Committee on Animal Experimentation (CETEA) of the Federal University of Minas Gerais, under the CEUA protocol: 333/2023.

### 2.2. Samples

This retrospective study used previously available paraffin blocks. Fragments with pre-analytical alterations, such as poor fixation and technical artifacts, were excluded. The most representative fragment of the tumor was selected for histopathological analysis and “hot-spots” with the following characteristics prioritized: the absence of autolysis, fibrosis, necrosis, and coalescent hemorrhage as well as weakly invasive carcinomatous areas in the stroma without myoepithelial delimitation. In cases with multiple fragments, the section with the highest presence of “hot-spots” was selected. For neoplasms larger than 5.0 cm, well-fixed formalin fragments without apparent necrosis or hemorrhage were recommended. Cases were divided into two groups: 25 cases of MTC at an early stage (T1,2,3N0M0) and 15 cases of MTC with lymph node metastasis (T1,2,3N1M0). All lymph nodes excised during surgery were subjected to histopathological evaluation.

Samples were collected at the Laboratory of Comparative Pathology of the Federal University of Minas Gerais and the Veterinary Pathology Laboratory of the University of Vila Velha. Most lesions were obtained from surgical excisions performed at the Veterinary Hospital of the Federal University of Minas Gerais (2010–2023).

### 2.3. Histology

Histological sections of 4 µm were obtained from fragments fixed in 10% neutral buffered formalin, processed for paraffin embedding, and stained by Hematoxylin–Eosin. Three pathologists evaluated the primary tumors based on the morphological characteristics described in Classification and Grading of Canine Mammary Tumors (2011) [1]. All cases analyzed in this article had a single diagnosis: MTC. The invasive areas were analyzed at 20×, 40×, and 60× magnification, and histomorphometry was performed. Histological subtypes were classified according to the WHO Classification of Tumours (2019) [13] and Classification and Grading of Canine Mammary Tumors (2011) [1]. The presence of metastases was evaluated in the regional lymph nodes [19,20,21].

### 2.4. Histomorphometry of Invasive Areas

The criteria for invasive areas included groups of infiltrative atypical epithelial cells in the stroma, the absence of myoepithelial cells, and the basement membrane. Microinvasive areas were defined as foci with a ≤1 mm diameter beyond the basement membrane. Photomicrographs were captured with an Olympus BX41 microscope and analyzed by ImageJ software (v.2.3.0). Measurements of area, perimeter, and length were converted to mm and mm^2^. Cut-off points were defined as 2 mm^2^ for the invasive area and 14 for the number of foci.

### 2.5. Immunohistochemistry

Slides with 4 µm sections of primary tumors were prepared and incubated with primary antibodies against ER, PR, HER-2, COX-2, and Ki67. Positive controls included fragments of canine mammary glands previously tested.

Immunoreactivity was visualized with DAB, and slides were counterstained with hematoxylin. Tumors were classified according to the following: immunophenotypes: luminal A: ER and/or PR positive, HER-2 negative, low Ki67 (<20%); luminal B HER-2 positive: ER and/or PR positive, HER-2 positive, any Ki67; luminal B HER-2 negative: ER and/or PR positive, HER-2 negative, Ki67 ≥ 20%; HER-2 overexpressed: ER and/or PR negative, HER-2 positive; triple-negative: ER, PR, and HER-2 negative [22,23]. The final semi-quantitative analysis of COX-2 was performed according to Lavalle et al. (2009) [24].

### 2.6. Specific Survival

Specific survival was defined as the time interval between the date of surgery and the occurrence of death or euthanasia specifically attributed to the progression of neoplastic disease. Patients whose deaths were unrelated to the neoplastic condition were excluded from the analysis. Follow-up data were collected and monitored up to March 2024.

### 2.7. Statistical Analysis

The Shapiro–Wilk test was used to assess data distribution. An unpaired T-test was performed to compare the number of invasive areas between MTC without metastasis and MTC with metastasis, and the Mann–Whitney test was used to compare the size, perimeter, and length of invasive areas between MTC without metastasis and MTC with metastasis. The Spearman Correlation Coefficient and linear regression statistical tests were used to establish correlations and associations between the size, perimeter, length, and number of invasive foci and tumor size, immunohistochemical markers Ki67, COX-2, ER, PR, and HER2, and immunophenotype. The chi-square test was used to correlate clinicopathological data with the parameters of grouped invasive areas, and data were represented in a contingency graph. Specific survival was estimated by a Kaplan–Meier curve, and comparisons between the number of invasive foci and the size, perimeter, and length of invasive areas were performed using the Mantel–Cox log-rank test. These analyses were conducted using Microsoft Windows and Prism software (version 7.0, GraphPad, San Diego, CA, USA). For all analyses, a *p*-value < 0.05 was considered statistically significant.

## 3. Results

### 3.1. Sample Characterization

Among the 40 cases of female dogs diagnosed with MTC, the age ranged from 7 to 20 years, with an average of 12.2 years. The most common breeds in the study included mixed-breed dogs, accounting for 22.5% (9/40) of the cases, poodles accounting for 17.5% (7/40) of the cases, and beagles accounting for 7% (3/40) of the cases, while information about breed was missing in 20% (8/40) of the cases. The most affected mammary glands were the caudal abdominal glands (30%) and the inguinal glands (27.5%). Non-ulcerated cases represented 77.5% (31/40) of the total, while 12.5% (5/40) of cases presented ulceration; 10% (4/40) of the cases lacked information on the presence or absence of ulceration.

Regarding the size of the primary masses evaluated macroscopically, 50% (20/40) were classified as T1 (<3 cm), 15% (6/40) as T2 (3–5 cm), and 30% (12/40) as T3 (>5 cm); size information was not available for 5% (2/40) of the cases. Regarding the presence or absence of metastases in regional lymph nodes, 37.5% (15/40) of the cases had metastases, while 62.5% (25/40) did not. Regarding the characterization of the molecular subtype of MTC with metastasis, Luminal A was the most frequent subtype, observed in 8 cases (53.3%), followed by Luminal B HER-2 positive in 6 cases (33.3 percent) and Luminal B HER-2 negative in 1 case (6.6%). In particular, no cases of HER-2 overexpression were identified.

In MTC without metastases cases, Luminal A represented 9 cases (36%), Luminal B HER-2 positive was observed in 6 cases (24%), Luminal B HER-2 negative was observed in 2 cases (8%), and no cases of HER-2 overexpression were found.

### 3.2. Evaluation of Size, Perimeter, and Number of Invasion Foci

**Figure 1:**
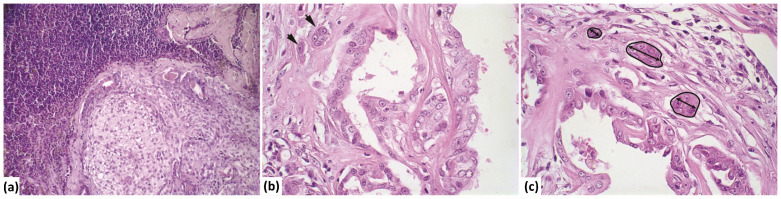
Photomicrograph of mixed-type carcinoma with metastasis. (**a**) Lymph node showing proliferation of atypical epithelial cells with a myxoid matrix partially replacing the lymphoid parenchyma (H&E stain, 20× magnification). (**b**) Invasion area (indicated by an arrow) with atypical epithelial cells infiltrating the stroma of the mixed-type carcinoma (H&E stain, 60× magnification). (**c**) Measurement of the invasion area, perimeter, number of invasion foci, and length (longest diameter) within the stroma of the mixed-type carcinoma (marked in black) (H&E stain, 60× magnification).

In female dogs with MTC without metastasis, the invasion areas ranged from 0.5 to 5.40 mm^2^. Conversely, this range varied from 0.3 to 33.9 mm^2^ in bitches with MTC with metastasis. The number of invasion foci in MTC without metastasis ranged from 1 to 15, while in MTC with metastasis, it ranged from 1 to 33 foci. A higher number of invasion foci were observed in MTC with metastasis compared to MTC without metastasis (Figure 1).

**Figure 2:**
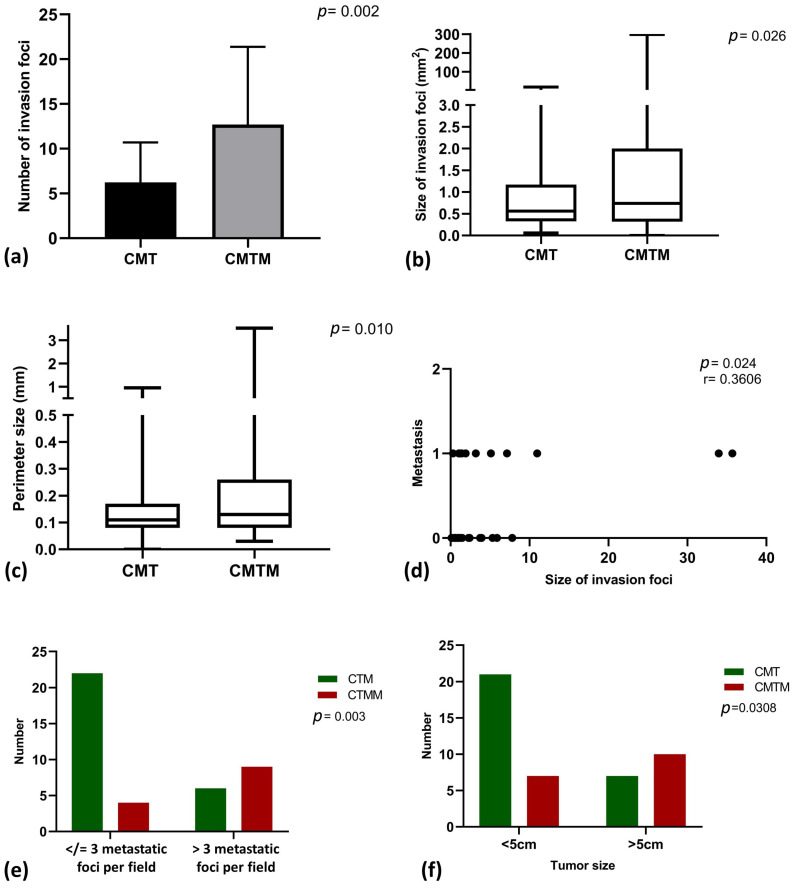
Assessment of the size, perimeter, and number of invasion foci in mixed-type carcinoma without metastasis (MTC) and mixed-type carcinoma with metastasis (MTCM). (**a**) Comparison of the number of invasion foci between mixed-type carcinoma without metastasis and mixed-type carcinoma with metastasis (unpaired T-test). (**b**) Comparison of the size of invasion foci between mixed-type carcinoma without metastasis and mixed-type carcinoma with metastasis (Mann–Whitney test). (**c**) Comparison of the perimeter of invasion foci between mixed-type carcinoma without metastasis and mixed-type carcinoma with metastasis (Kolmogorov–Smirnov test). (**d**) Correlation between lymph node metastasis (coded as 0 for mixed-type carcinoma without metastasis and 1 for mixed-type carcinoma with metastasis). (**e**) Association between the number of invasion foci per field and the presence of lymph node metastasis. (**f**) Association between tumor size and the presence or absence of lymph node metastasis.

The comparison of the number of invasion foci between MTC with metastasis and MTC without metastasis is illustrated in Figure 2a. The number of invasion foci was significantly higher in MTC with metastasis compared to MTC without metastasis. Figure 2b presents a comparison of invasion focus size between the two groups, showing that the size of invasion foci is also larger in MTC with metastasis than in MTC without metastasis. Additionally, the perimeter of the invasion areas was found to be greater in MTC with metastasis compared to MTC without metastasis (Figure 2c).

A moderate direct correlation was observed between the size of the invasion areas and the presence of lymph node metastasis (Figure 2d). MTC cases with metastasis (coded as 1) showed larger invasion areas compared to MTC cases without metastasis (coded as 0). Figure 2e highlights the association between the size of invasion foci and the presence of lymph node metastasis. Most MTC cases without metastasis presented invasion foci smaller than 3 mm^2^, while the majority of MTC cases with metastasis exhibited invasion foci larger than 3 mm^2^.

Figure 2f highlights the association between the tumor size and the presence of lymph node metastasis. Most mixed-type carcinoma without metastasis cases presented invasion foci smaller than 3 mm^2^, while the majority of mixed-type carcinoma with metastasis cases exhibited invasion foci larger than 3 mm^2^. Figure 2f shows the relationship between the number of invasion foci per field and the presence of lymph node metastasis. Tumors smaller than 5 cm were primarily associated with the absence of metastasis, whereas tumors larger than 5 cm were predominantly associated with lymph node metastasis.

Comparisons, correlations, and associations were performed between microinvasion areas, invasion lengths, immunophenotype, COX-2 immunoexpression, and the cell proliferation rate between MTC cases without and MTC with metastasis. However, no statistically significant differences were observed.

### 3.3. Evaluation of the Impact of Lymph Node Metastasis, Size, and Number of Invasion Foci on Specific Survival

**Figure 3:**
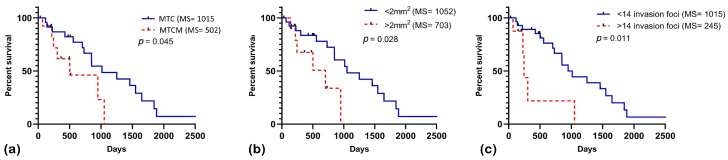
Kaplan–Meier survival curve for the specific survival of patients diagnosed with mixed-type carcinoma. (**a**) The mixed-type carcinoma with metastasis group (MTCM) reached the median survival on Day 1015, while mixed-type carcinoma without metastasis (MTC) group reached it on Day 502. (**b**) Patients with invasion areas smaller than 2 mm^2^ reached the median survival on Day 1052, while those with invasion areas larger than 2 mm^2^ reached it on Day 703. (**c**) Patients with more than 14 invasion foci had a median survival of 245 days, while those with fewer than 14 invasion foci reached the median on Day 1015.

To assess the influence of regional lymph node metastases on specific survival, the survival times of dogs diagnosed with MTC without metastasis and MTC with metastasis were compared. Dogs diagnosed with MTC without metastasis showed a significantly longer specific survival time (1015 days) compared to those diagnosed with MTC with metastasis (502 days) (Figure 3a) (*p* < 0.05). Regarding the number of invasion foci, patients with fewer than 14 foci reached a median survival of 1015 days, while those with more than 14 foci had a median survival of 245 days (Figure 3b).

For the size of the invasion areas, dogs with invasion areas smaller than 2 mm^2^ reached a median survival of 1052 days, while those with areas larger than 2 mm^2^ reached a median survival of 703 days (Figure 3c) (*p* < 0.05). It is important to note that the cut-off point of 2 mm^2^ was determined by different statistical tests. While the 3 mm^2^ cut-off was established for association analyses, survival correlation was assessed by the Kaplan–Meier test. Additionally, some data were excluded from this analysis due to incomplete follow-up records, which may have influenced the defined cut-off points.

The perimeter size, invasion length, immunostaining of hormone receptors, HER-2 and COX-2, cell proliferation rate, and microinvasion areas of the cases did not have a statistically significant impact on patient survival.

## 4. Discussion

Canine mammary tumors are the most common neoplasm in female dogs, with MTC being the most frequent histological type. While generally associated with a more favorable prognosis than other malignant types [1,2,3,4,5,6,7,8,9,25,26], MTC can exhibit invasive behavior and metastasize regionally, impacting prognosis [11,12]. The Nottingham system, widely used in human breast cancer, is recommended for evaluating the invasive component of canine mammary carcinomas [16]. This system considers tubular formation, nuclear pleomorphism, and mitotic count. However, its application to MTC can be challenging due to limited invasive areas, particularly microinvasion, which complicates accurate mitotic counts and histological grading. These challenges can directly affect prognostic estimation and treatment planning [14,15].

This study investigates MTC, comparing groups with and without regional lymph node metastases, and evaluating the number and size of invasive carcinomatous areas. Our results demonstrate that the number and size of invasive areas are significantly associated with metastasis and reduced survival in dogs with MTC [22]. While previous research on ductal carcinoma in situ with microinvasion suggests that the number of microinvasive foci alone may not directly influence prognosis, further investigation is needed to clarify the role of these areas in tumor behavior [14,15]. It is important to note that the quantification of invasive areas in 2D is not entirely unbiased, as this technique has known limitations. It does not provide data on the entire tissue and relies on certain assumptions. Stereological methods, on the other hand, can obtain 3D information, offering a more precise approach for analyzing histomorphometry in tissue samples [27]. Future studies should focus on these methods and compare their results with the 2D data presented in this article.

The present study found a greater number of foci and larger invasive areas in the group with metastases compared to the group without metastases. A direct, moderate correlation was observed between the size of the invasive area and the presence of regional lymph node metastases, highlighting the importance of these characteristics for prognosis. The MTC without metastasis group predominantly presented with fewer than three invasive foci and areas smaller than 3 mm^2^, while the MTC with metastasis group more frequently exhibited more than three foci and areas larger than 3 mm^2^. These findings are consistent with the established importance of lymph node status as a crucial prognostic factor in both canine and human mammary cancers, as regional lymph node metastasis is associated with reduced survival [19,20,22,25,26,28,29].

The perimeter of invasive areas was also larger in the MTC with metastasis group, though not directly associated with survival. This may reflect the unique biology of MTC, potentially related to the role of collagen fibers in the tumor microenvironment. Studies suggest that reduced linearity and length of stromal collagen fibers may facilitate tumor invasion, potentially increasing interaction between tumor cells and the microenvironment [22,26,30,31,32,33].

Immunophenotypic analysis revealed Luminal A and Luminal B HER-2 positive as the most frequent subtypes in both groups, with no statistically significant association with metastasis. This suggests that other factors, like tumor size and the number of invasion foci, may be more influential in predicting MTC metastasis. However, the literature indicates that dogs with Luminal B HER-2 positive mammary carcinomas have shorter survival times [23,34,35,36,37], emphasizing the need for further research on the role of molecular profiles in mixed tumors. Although no significant association was found between immunophenotypes and metastasis in this study, the expression of Ki67, ER, PR, COX-2, and HER-2 could still influence MTC behavior. For example, Ki67, a cell proliferation marker, may be associated with an increased risk of local recurrence or distant metastasis, even without a direct correlation with lymph node involvement [17,18,23,24].

Tumor size is a well-recognized independent prognostic factor in canine mammary neoplasms [2,5,7,8,28]. In this study, most cases with lesions larger than 5 cm had lymph node metastasis, while those smaller than 5 cm generally did not. Dogs with tumors larger than 5 cm (T3) have shorter survival times than those in T1 and T2 stages. The largest tumor diameter is used for clinical staging. Clinical features like rapid tumor growth, signs of invasiveness, and inflammatory conditions are also important and have been associated with poorer prognosis [2,5,7,8,28,29]. Cavalcanti et al. observed that, in MTC, the size of the invasive malignant epithelial area negatively impacts survival, with larger areas associated with shorter survival [6]. This aligns with our findings, where the number and size of invasive areas directly affected survival.

Finally, this study found a significant reduction in specific survival in dogs with MTC with metastasis compared to MTC without metastasis. The median survival was 502 days in the MTC with metastasis group and 1015 days in the MTC without metastasis group. These results are consistent with previous studies, demonstrating that the size of the invasive malignant epithelial area directly influences survival, with a larger size and more foci associated with worse outcomes [6].

## 5. Conclusions

The number of foci and the size of the invasive areas in the stroma of MTC are crucial prognostic factors in female dogs, which influence both the risk of metastasis and survival. These findings underscore the necessity for early detection and detailed histopathological characterizations to guide more effective therapeutic strategies, such as surgical removal of the tumor and lymph nodes, as well as adjuvant therapies like chemotherapy and radiation therapy. Future studies should focus on elucidating the impact of microinvasive areas and their interactions with the tumor microenvironment, aiming to deepen our understanding of tumor progression and aggressiveness in this unique carcinoma subtype. Additionally, further research should explore the molecular mechanisms underlying the development and progression of MTC, which could lead to the identification of new therapeutic targets and the development of more effective treatments.

## Data Availability

The data presented in this study are available from the corresponding author upon request.
